# Improving public health service delivery response to address contraceptive needs of socio-economically disadvantaged HIV positive people in Maharashtra, India

**DOI:** 10.1186/s40834-021-00159-4

**Published:** 2021-05-03

**Authors:** Beena Joshi, Bhushan Girase, Siddesh Shetty, Vinita Verma, Shrikala Acharya, Pramod Deoraj, Ragini Kulkarni, Shahina Begum

**Affiliations:** 1 Indian Council of Medical Research- National Institute for Research in Reproductive Health (NIRRH), Mumbai, Maharashtra India; 2 Family Health Department, PATH India, New Delhi, India; 3 National AIDS Control Organization, New Delhi, India; 4 Mumbai District AIDS Control Society, Mumbai, Maharashtra India; 5 Maharashtra State AIDS Control Society, Mumbai, Maharashtra India

**Keywords:** Dual contraception, HIV-FP linkage, Women living with HIV, Unmet need for contraception, Unintended pregnancy, Prevention of parent to child transmission

## Abstract

**Background:**

People living with HIV (PLHIV) receive free antiretroviral treatment (ART) in public health facilities of India. With improved life expectancy, unmet sexual and reproductive health needs of PLHIV have to be addressed through a converged programmatic response strategy. Evidence shows that socioeconomically disadvantaged women are most vulnerable to high reproductive morbidities, especially HIV positive women with an unmet need of contraception.

**Methods:**

Programmatic convergence by linking ART and family planning services were strengthened at two public health facilities (district hospitals) generally accessed by disadvantaged socio-economic sections of the society. Barriers to linking services including stigma and discrimination were addressed through analysis of existing linkage situation, sensitization and training of healthcare providers and system-level interventions. This facilitated provider-initiated assessment of contraceptive needs of PLHIV, counseling about dual contraception using a couple approach, linkage to family planning centers and maintaining data about these indicators. Six hundred eligible PLHIV seeking care at ART centers were enrolled and followed up for a duration of 6 months. Acceptance of family planning services as a result of the intervention, use of dual contraception methods and their determinants were assessed.

**Results:**

Eighty-seven percent HIV couples reached FP centers and 44.6% accepted dual methods at the end of the study period. Dual methods such as oral contraceptive pills (56.2%), IUCDs (19.4%), female sterilization (11.6%), injectable contraception (9.9%) and vasectomy (2.9%) in addition to condoms were the most commonly accepted methods. Condom use remained regular and consistently high throughout. The study witnessed seven unintended pregnancies, all among exclusive condom users. These women availed medical abortion services and accepted dual methods after counseling. Female index participants, concordant couples, counseling by doctors and women with CD4 count above 741 had higher odds of accepting dual contraception methods. Standard operating procedures (SOP) were developed in consultation with key stakeholders to address operational linkage of HIV and family programs.

**Conclusion:**

The study saw significant improvement in acceptance of dual contraception by PLHIV couples as a result of the intervention. Implementation of SOPs with supportive supervision can ensure efficient linkage of programs and provide holistic sexual and reproductive healthcare for PLHIV in India.

## Background

Human Immunodeficiency Virus (HIV) infection remains a major global public health concern with 38 million infected individuals and 1.7 million new cases in the year 2019 [1]. An estimated 1.3 million pregnant women in the world lived with HIV, exposing them to the risk of maternal to child transmission [2]. The disease is successfully suppressed by the highly effective antiretroviral therapy and is recommended for all People Living with HIV (PLHIV) including pregnant and breastfeeding women. A four-pronged strategy for Prevention of Parent to Child Transmission (PPTCT) namely preventing HIV infection in women of childbearing age, preventing unintended pregnancies in HIV infected women, preventing transmission of HIV from infected mother to her infant by drug therapy and providing appropriate treatment, care and support to the needs of the mother, child and family are outlined by the World Health Organization (WHO) [3].

India has managed to reverse the HIV epidemic owing to its phased national programs. The Indian national program provides direct access to free diagnostics, free Anti-Retroviral Therapy (ART) therapy, PPTCT services, management of opportunistic infections including tuberculosis (TB) all through a single-window approach. Consequently, the country has contributed to global success by making significant national gains in reducing new HIV infections by more than 60% since the year 2000 and 71% reduction in Acquired Immunodeficiency Syndrome (AIDS) related deaths since the year 2005 [4]. At an adult HIV prevalence of 0.22%, India had an estimated 22,677 (10927–40,605) pregnant women living with HIV in the year 2017 [4]. India is signatory to the 90:90:90 United Nations (UN) strategy and aims to end HIV/AIDS by the year 2030. The country has prioritized elimination of mother to child transmission as one of its immediate objectives to achieve HIV elimination [5]. India has adopted the WHO four-pronged strategy for its federally implemented PPTCT program, but efforts have primarily focused on the third prong i.e. preventing transmission from mother to child using Option B plus prophylaxis. As a result, the opportunity of preventing unintended pregnancy itself is missed, increasing the investment burden of resources needed for third prong of the program [6]. Avoiding unintended pregnancies among the immuno-compromised PLHIV population presents a potential opportunity to safeguard their sexual and reproductive health (SRH) rights. Comprehensive access to contraception services facilitate PLHIV to plan their family desires, reduces transmission of sexually transmitted infections (STI) and helps getting screened for reproductive health morbidities. This benefits the larger population health outcomes. For women, contraception specifically offers control of reproduction cycle, helps delay first birth, improves spacing, reduces total number of children borne and prevents high-risk and unintended pregnancies thus also reducing the need for unsafe abortions. Preventing unintended pregnancy gains added significance as it can potentially reduce number of infants born with HIV or children getting orphaned as a result of losing their parent [7]. The current consensus suggests HIV infected women can use any of the available modern contraceptive methods [8]. Using condom alone prevents STIs and pregnancy but comes with a drawback of a high failure rate as a contraceptive and is not a woman-controlled method.

Global advocacy agenda has shifted to implementation of interventions addressing combined HIV and SRH needs most recently stated by the 2018 ‘Call to Action’ for attaining universal health coverage [9]. Consistent and correct use of male or female condoms supplemented by other methods is endorsed for providing contraception services in context of HIV treatment programs [10]. This becomes crucial for India as it has an unmet need for family planning (FP) at 13% in the general population with a higher reported need of 17% in PLHIV group [11, 12]. Further, women’s socioeconomic status could have an association with their sero-status, as HIV-positive women are significantly more likely to have low levels of education than their HIV-negative counterparts [13]. Association of socio-economic determinants like age, education, place of residence along with gender-based discrimination and domestic violence faced by Indian women make them vulnerable to both HIV and unintended pregnancies. The current HIV/AIDS service delivery in India typically focuses on condoms as the method for preventing spread of STIs and HIV but fails to acknowledge strengths of dual methods of contraception. FP service provided by the Indian PPTCT program is limited to providing general information regarding contraception as and when demanded by PLHIV. There is no active assessment of unmet contraception need or provider-initiated referral to FP centers. Management Information System (MIS) is without indicators for reporting and monitoring of these measures. Inequity in access to healthcare services further poses challenges as a result of HIV related stigma and discrimination or lack of proper knowledge among healthcare providers about services that can or cannot be provided to HIV positive people. Healthcare providers are expected to play an integral role in identifying and meeting sexual and reproductive health needs of this marginalized population. Non-HIV related service providers often are not sensitized to their needs, have misconceptions or sometimes fear about the iatrogenic spread of infection [14, 15]. Their personal belief sometimes leads to unfriendly attitudes resulting in delaying or refusal of treatment [16]. This results in poor health-seeking behavior and reduces access opportunities to the already disadvantaged sub-groups like low socio-economic sections of this vulnerable population.

This study aimed at upscaling a pretested intervention at district hospital settings to improve system level efficiency and effectiveness of linkage between HIV and family planning services with a focus on dual contraception to prevent unintended pregnancies among PLHIV. The objectives of this study included assessment of the current situation of linkage between HIV and family planning services, assessment of knowledge, attitude and practices of contraception among PLHIV, implementation of the pretested intervention linkage package with modifications based on situation analysis and assessment of post intervention acceptance of dual methods of contraception and its determinants among PLHIV.

## Methods

This implementation study was conducted between April 2016 to May 2018. The up-scaling of this implementation research was framed based on experience from a previous study undertaken by the authors that focused on linking HIV and family planning services and promoting use of dual contraception among PLHIV at two tertiary care centers in Mumbai, India between 2011 and 2013. The study conducted among 300 HIV positive women had demonstrated improved uptake of dual methods without reduction in consistent use of condoms. Fewer unintended pregnancies in the experiment group were also reported [17, 18]. The program implementation agency i.e. NACO recommended implementation of this study to be undertaken at district level settings. Two district hospitals namely Civil hospital, Parbhani and District hospital, Aundh, Pune from the state of Maharashtra in India were chosen as the sites for this study after fulfilling criteria such as patient load at ART center, availability of staff at facility and considering operational feasibility. Ethical approvals from requisite institutions and administrative approvals from the chosen hospitals were obtained before initiating the study. Privacy and confidentiality of participants was ensured throughout. An informed consent was obtained from PLHIV before enrollment to the study.

### Situation analysis of HIV-FP linkage

Healthcare providers and program managers were interviewed to assess the existing linkage status and estimate training needs to facilitate provision of linked services. A total of seven medical doctors, six counselors and two nursing staff from the study sites were selected as key informants based on convenience sampling strategy and were interviewed using a semi-structured interview schedule adapted from an internationally developed rapid assessment tool for SRH and HIV linkage [19]. Participatory observations, facility survey checklists and informal interaction with program managers were additionally used to assess physical infrastructure, referral linkage and availability of Information, Education and Counseling (IEC) materials and supplies at the study sites. Details and methods used to assess provider perspective is also published elsewhere [20].

### PLHIV enrollment and baseline assessment of knowledge, attitude and practices of contraception

HIV positive couples at ART centers of the two chosen district hospitals were screened for eligibility of acceptance of dual contraception methods. HIV concordant or discordant couples (women aged 18–40 years) not having undergone permanent sterilization and using only condoms as a contraceptive method, if did not wish pregnancy for a year and were willing to follow-up for 6 months were considered eligible for the study. An estimated sample size of 246 couples from each centre i.e. 492 couples from the two district hospitals was calculated based on average statistics of PLHIV attending HIV facilities and considering 20% increase in acceptance of dual methods [17]. Further assuming a loss to follow-up, the estimated sample size was increased to 600 couples. A total of 2663 PLHIV clients visited the chosen ART facilities during the study period. Based on the pre-defined inclusion and exclusion criteria for participation, a successive/consecutive sampling technique was used to enroll 259 index participants at Pune site and 311 participants at Parbhani facility, thus enrolling 570 eligible couples for the study after obtaining the written informed consent. Age criteria, permanent sterilization, marital status (separated/divorced/widow) or pregnancy at enrollment were the commonest causes for exclusion of cases. Among those enrolled, there were four deaths (three male and one female index participant) due to tuberculosis, one couple refused further participation, five couples separated during the study course, one woman underwent hysterectomy for dysfunctional uterine bleeding and two were lost to follow-up. Eventually, data of 556 eligible couples, 256 from Pune and 300 from Parbhani facility completing all follow-ups were considered for analysis. This number was over and above the expected sample size of 492 due to fewer losses to follow-up.

A pretested semi-structured interview schedule divided into two sections with the first section administered at enrollment to collect information on study participants and at followup. Data on socio-economic status, contraception use, sexual practice, fertility desires, reproductive health knowledge and psychological well-being of the individuals was obtained at enrollment.

### Intervention package, implementation challenges and actions taken

The intervention focused on providing linked HIV and FP services and promoting dual contraception among enrolled couples at designated healthcare facilities of chosen district hospitals. The following health system interventions were strengthened to fill gaps identified by situation analysis and stakeholder engagement.

- Healthcare providers at both HIV and FP centers were trained to impart desired skills for service provisioning. Workshop using lectures, group discussions, role plays, brainstorming of case studies were used to impart knowledge and skills of counseling techniques and desired service provisioning. Midterm refresher training was conducted to sensitize providers. Orientation and planning meeting were held with key stakeholders to operationalize the linkage strategy.
- Information, Education and Counseling (IEC) strategies were developed to improve awareness on HIV, STI, FP and modern contraception. Posters and pamphlets of dual protection, prevention of unintended pregnancies (UIP), FP methods, information on medical eligibility criteria for contraceptive use, pregnancy checklist, etc. by adapting government guidelines for providers were developed in local language of the study sites.
- Provider-initiated assessment of unmet need for contraception were established at ART centers by training counsellors for assessment of eligibility for contraception use as per WHO eligibility criteria, identification and assessment of needs and determinants of unmet contraception, clinical assessment and importance of provider-initiated referrals.
- Eligible PLHIV were referred from ART center to FP center using referral slips as a substitute to existing oral referrals.
- A couple approach was used to counsel eligible PLHIV on dual methods of contraception along with correct and consistent use of condoms.
- Management Information Systems (MIS) was maintained at both sites to capture referrals from ART centers, FP services attendance of referred cases and acceptors of dual methods.
- Enrolled PLHIV were followed up bi-monthly during their routine medication visit to ART center.
- Convergence and cross talks were facilitated between HIV and FP center staff. These interactions helped chalk out constraints faced by providers in implementation and identify solutions for the same.
- Many challenges were faced during implementation of the intervention package. This included supply-side challenges such as apprehensive attitude of providers, undue referrals to tertiary facilities and stock-out of FP commodities and drugs. Attitudinal issues comprised discrimination of PLHIV by delaying consultations, counseling or advising unwarranted pathophysiological investigations with associated surgical procedures. Table 1 summarizes the actions taken against broad challenges faced in implementation of the intervention.

**Table 1:** Challenges faced during implementation of the study and corresponding actions undertaken

Activity	Challenges	Action Taken
**HIV-FP linked services**	**Discriminatory services and negative work attitude of providers**	▪ Brainstorming and experience sharing with health providers regarding their apprehensions about providing services to people living with HIV (PLHIV) ▪ Refresher training to guide district-level health providers in assessing medical eligibility of PLHIVs for contraceptive use ▪ Advised health providers to follow universal safety measures while performing IUCD insertion, medical termination of pregnancy (MTP), Tubal Ligation (TL) and delivery of HIV positive women
**Supplies**	**Inopportune indenting and frequent stock-out**	▪ Liaised with the district, regional and state officials of Family Welfare Bureau and State AIDS Control Society in issuing required FP commodities and STI kits ▪ Supplied Information Education Counselling (IEC) materials on FP and dual methods of contraception
**Management Information System**	**No/under-reporting of FP indicators**	▪ Strengthened monitoring to maintain records of HIV-FP linked services at Obstetrics and Gynecology (OBGY) and Out-Patient Department (OPD) ▪ Conducted concurrent meetings with concerned health providers at ART, ICTC, and STI centers to report FP related indicators in monthly reporting formats

### Assessment of uptake of dual methods post intervention

The couples after initial enrollment and counselling at ART center were referred to FP centers using referral slips and facility-level linkages. Provisioning of accepted dual methods was done at the FP center. Enrolled PLHIV couples were then followed up bimonthly for up to six consecutive months at ART centers for follow-up counseling, assessment of acceptance and uptake of dual contraception. Section two of the semi-structured interview schedule was designed to assess and keep track of the respondent’s health status, sexual activity, change in fertility desire, menstruation if applicable, any pregnancy or abortion, reasons for non-use of contraception and challenges faced in accessing contraception services.

#### Data analysis

SPSS Version 19 (SPSS Statistics for Macintosh, Version 19.0 (Armonk, NY: IBM Corp) was used for data analysis. Open-ended answers were coded. Categorical variables are presented as numbers, percentages and means. Bivariate comparisons were analyzed using a chi-squared test for categorical variables. Logistic regression analysis was run to assess determinants associated with acceptance of dual contraception methods. The adjusted Odds Ratio (OR) and 95% Confidence Interval (CI) was calculated and is presented in the results section.

## Results

### Existing HIV-FP linkages status

The ART centers were providing testing, counseling, free antiretroviral drug distribution and support services as mandated. Though condom promotion was encompassed by free distribution, demonstration of its use was demand specific. Nearly half the provider respondents (58% at Pune and 44% at Parbani) were engaged in providing FP services and post-abortion counselling. Male clients were not allowed to enter obstetrics-gynecology out-patient department (OPD) and female clients seeking FP advice were neither provided condoms nor were counseled on its correct use. Nearly 80% respondents from FP centers revealed that no mechanism for follow-up of referral cases existed. Existing referrals were exclusively for sero-positive pregnant women unless a specific service was demanded.

The respondent workforce was academically qualified, experienced and trained during induction as mandated but desired refresher training. Half the key informants presumed condom use alone to be sufficient to prevent pregnancies. Nearly half (47%) providers perceived contraceptives to be harmful or contra-indicated for PLHIV. Most respondents were unaware of mother-to-child transmission rate at their respective facilities.

Indenting and supply of FP commodities was irregular, inopportune and dependent on demand at facilities. Oral Contraception (OC) pills despite being available at pharmacy of the district hospitals was not supplied at respective FP facility. Emergency contraceptives were unavailable at both hospitals. The district hospitals were well-equipped for IUCD and permanent sterilization. IEC material on FP services for PLHIV was limited to only condom promotion with complete lack of materials for dual methods of contraception.

The ART facilities maintained patient and program monitoring records periodically as mandated. Though patient card had provisions for recording contraception and obstetric history, these were not updated for FP indicators including number of condoms distributed at the facility. The reproductive health facilities at district hospitals maintained registers for OPD, IUCD, antenatal clinics and high risk pregnancy but did not list indicators pertaining to FP service such as method advised by provider, method accepted by client, reason for any method ineligibility, follow-up date and reason for discontinuation of a given method. Moreover, seropositive status of FP acceptors at FP facility was not recorded.

### PLHIV characteristics and health status at enrollment

At enrollment, the mean age of men and women participants was 36.16 (±6.3) and 30.15 (±5.3) years respectively. Women accounted for 43.3% (241/556) of all index participants accessing ART services. Nearly one-third (165/556) participants, predominantly from Parbhani district (158/165) resided in rural areas. Nearly 19 % (105/556) participants resided in urban slums and remaining 51.3% (286/556) were from urban localities. Based on the modified Kuppuswamy scale for socio-economic classification-2019 used in Indian context, more than 93% (518/556) couples belonged to either lower-middle or lower socio-economic categories [21]. Only 3.6% (20/556) females in the study cohort as compared to 38% (212/556) males were in skilled or semi-skilled occupations. Nearly 90% (501/556) females were unskilled workers as compared to 35.9% (200/556) unskilled male counterparts. Illiteracy was more common in females with 16% (89/556) illiterate women as compared to 12.4% (69/556) illiterate males in the cohort. Nearly 82% (456/556) PLHIV couples lived in nuclear families. A mean family size of 3.96 (±1.34) members and mean 1.64 (±1.06) children in the family were reported. A monthly median household income of Indian Rupee (INR) 8000 (United States Dollar (USD) 117) [Interquartile Range (IQR) INR 5000–12,000 (USD 73–175)] was reported as per the 2018 currency exchange rate [22]. Median per capita income was INR 2500 (USD 37) [IQR INR 1549–4000 (USD 23–58)]. Table 2 enlists socio-demographic characteristics of all participants that completed the study from two chosen district hospitals of Maharashtra, India.

**Table 2:** Socio-demographic characteristics of PLHIV respondents

Variable		Percentage (%) (***n*** = 556)	
**Age**		Male	Female
	19–25	3.4	24.3
	26–32	27.5	42.8
	33–40	49.3	30.8
	41–45	19.8	2.2
**Educational status**	Illiterate	12.4	16.0
	Primary & Middle	24.3	34.7
	High school & intermediate	48.4	41.9
	Graduate	8.8	4.9
	Professional degree	6.1	2.5
**Occupation**	Skilled & semi-skilled	37.9	3.6
	Unskilled	36.0	90.1
	Professional or semi-professional	2.7	1.4
	Business, farmer, clerical	22.8	4.7
	Unemployed	0.5	0.2
**Place of residence**	Urban slum	18.9	
	Urban non-slum	51.3	
	Rural	29.7	
**Socio-economic classification of the family**	Modified Kuppuswamy classification, 2019		
	Upper	0.0	
	Upper-middle	6.8	
	Lower-middle	46.9	
	Upper-lower	45.0	
	Lower	1.3	

Almost 99% (549/556) index participants had disclosed HIV status to their spouse. The study comprised 55% (306/556) concordant and 42.4% (236/556) discordant couples. The remaining 2.5% (14/556) participants did not know HIV status of their spouse. Participants had HIV for a mean duration of 4.14 (±3.27) years. Of the total 556 couples who completed the study, 88.3% (492/556) women had children, with 9.9% (49/492) women having one or more HIV infected children. Thirteen percent (75/556) index participants had experienced symptoms of Reproductive Tract Infection/Sexually Transmitted Infection (RTI/STI) of which only one third i.e. 36% (27/75) had sought treatment. Obstetric history revealed that 28% (156/556) women had previously undergone Medical Termination of Pregnancy (MTP). The risk of fear of HIV to fetus and unwanted pregnancy accounted for being the commonest reason for women diagnosed with HIV undergoing an abortion.

Overall knowledge about safe sexual practice was limited to only use of condoms during sexual activity with 98.2% (547/556) participants aware of this practice. Nearly one-third i.e. 29.5% (164/556) were aware of avoiding risky sexual partners. Nineteen percent (106/556) participants were aware of avoiding multiple sexual partners. Awareness about use of methods of contraception was highest for male condoms at 98.2% (554/556), followed by female sterilization at 89% (495/556), IUCD at 61.7% (343/556), OC pills at 56.5% (314/556) and vasectomy at 51.9% (289/556). Only 2 % (11/556) participants were aware of female condoms as a method of contraception. Knowledge of dual protection was again restricted to only use of condoms. Nearly 73% (406/556) participants were aware of use of condoms to prevent pregnancy outcomes, HIV, and STIs. None of the participants had knowledge of using two methods i.e. a more effective method in addition to condoms for dual protection. Nearly 90% (500/556) participants believed condoms provided 100% protection against pregnancy. Nearly 16 % (87/556) participants believed that contraception was harmful to PLHIV. Of these, 72.4% (63/87) perceived IUCDs to be harmful for reasons such as perforation and other associated side effects. OC pills were perceived to be harmful by 25.3% (22/87) participants for reasons like abdominal pain, irritation, weakness and need for daily medication intake. Decision making for use of contraception was dependent most commonly on the doctor in 42.7% (237/556) cases, followed by choice of the sexual partner in 39% (217/556) cases and other family members in case of 3.9% (22/556) participants. Awareness about drugs and other treatment provision availability for preventing parent to child transmission of HIV was observed in 58% (323/556) participants.

Contraception use history revealed 11.3% (63/556) participants to have used modern methods of contraception before knowing their HIV status. None of the participants had previously used dual methods of contraception. Eighty-eight percent (492/556) participants did not desire children. Completed family in 49.7% (245/492) cases, risk of HIV transmission to child among 38.8% (191/492) were the commonest reasons given for not desiring children. Government facilities were used by 52.8% (294/556) participants to access condoms. Remaining 47.2% (249/556) sought pharmacies (246/249) or other Non-Governmental Organizations (NGOs) to avail it. Counseling at ICTC centers involved demonstration of correct use of condoms in 64% (356/556) participants previously.

### Impact of intervention

The intervention package resulted in a significant improvement in participant’s knowledge of contraceptives [from a baseline score of 3.84 to post-intervention score of 6.72] (highest score being 8) and safe sex practice [from a baseline score of 1.54 to 3.25 post-intervention] (highest score being 4). Awareness of using condoms in spite of both partners being HIV positive improved from 86.3% (480/556) to 100% by end of the study.

The intervention package resulted in 87% of participants reaching FP centers after being referred from ART center. Acceptance of dual contraception methods improved over time with 44.6% (248/556) participants accepting dual contraception methods by end of the study. The most preferred method in addition to condoms was OC pills followed by IUCDs, tubectomy, Injectable, and vasectomy. Noticeable turn-around time for acceptance of dual contraception methods was seen with 50% (124/248) acceptors accepting dual methods within first follow up after referral. Condom use among sexually active participants remained steadily high with 96.16% (526/547) participants regularly using condoms at end of the study period. Table 3 shows the type of dual contraception method in addition to condoms accepted and continued by participants until the end of the study period.

**Table 3:** Type of dual contraceptive method accepted and continued during the study period

Characteristic	Total (%)
**Method initially accepted along with condom (dual method)**	(*n* = 242)
Oral Contraceptive pills	136 (56.2%)
IUCD	47 (19.4%)
Injectable	24 (9.9%)
Tubectomy	28 (11.6%)
Vasectomy	7 (2.9%)
**Method used at the end of the study period along with condom**	(*n* = 248)
Oral Contraceptive pills	131 (52.8%)
IUCD	55 (22.2%)
Injectable	14 (5.6%)
Tubectomy	39 (15.7%)
Vasectomy	9 (3.6%)
**Regular condom use among sexually active participants over time**	(*n* = 541/547)
At Enrollment	512/547 (93.6%)
1st Follow Up	526/547 (96.2%)
2nd Follow Up	524/547 (95.8%)
3rd Follow Up	526/547 (96.2%)

Participants reported a change in fertility desire over course of the study period. Fifteen women reported pregnancy. Eight women had planned pregnancies. Seven unplanned pregnancies were reported. All seven unplanned pregnancies occurred among non-acceptors of dual methods i.e. only using condoms as the method of contraception. Six of these seven women underwent medical termination of pregnancy whereas one case reported a spontaneous abortion. These women were counseled post-abortion which resulted in four women accepting OC pills, one accepting an IUCD, one continuing with condoms, and one couple opting for vasectomy. Discontinuation was observed for OC pills (30 cases), three monthly Injectable (12 cases), and IUCDs (1 case) among dual methods. Switching of the dual method was also seen, commonly for OC pill acceptors (24 cases), injectable users (4 cases) and IUCD acceptors (3 cases). Table 4 shows acceptance, switching, and discontinuation of dual methods among participants in the study.

**Table 4:** Dual method acceptance, switching and discontinuation of methods among participants in the study

Type of FP Methods	FP method accepted in addition to condom as the dual method by end of the study	Method switching during the Study	Discontinued at study completion
Oral Contraceptive pill	131	24	30
Intra Uterine Contraceptive Device	55	3	1
Tubal Ligation	39	–	–
Injectable	14	4	12
Non-Scalpel Vasectomy	9	–	–
Total (N)	248	31	43

### Determinants of dual contraception acceptance

Logistic regression analysis was run to assess determinants of acceptance of dual methods by couples. Female index participants [OR 2 (99% CI: 1.42–2.81)], concordant couples [OR 1.97 (99% CI: 1.4–2.77)], decision making enabled by counseling provided by doctor [OR 1.63 (95% CI: 1.04–2.53)], CD4 counts above 741 [OR 1.7 (95% CI: 1.07–2.71)] were cases more likely to accept dual methods of contraception in study group apart from the ones who preferred method other than condoms [OR 4.92 (99% CI: 3.26–7.44)]. Table 5 describes these findings of determinants of dual contraception acceptance.

**Table 5:** Determinants of dual contraception acceptance

Background Variables		Unadjusted Odds Ratio	Adjusted Odds Ratio
**Index client**	Male	1	1
	Female	2** (1.42–2.81)	1.66* (1.12–2.46)
**Education Status**	Literate	1	1
	Illiterate	1.62 (0.96–2.72)	1.44 (0.8–2.59)
**CD4 count above 80th Percentile**	No	1	1
	Yes	1.26 (0.83–1.9)	1.7* (1.07–2.71)
**Partner HIV Status**	Negative	1	1
	Positive	1.97** (1.4–2.77)	1.65* (1.13–2.43)
**Decision Making**	Self	1	1
	Husband/Family/Doctors/Others	1.63* (1.04–2.53)	1.26 (0.77–2.08)
**Method preference**	Condom Only	1	1
	Dual methods	4.92** (3.26–7.44)	4.56** (2.94–7.06)

**p < 0.001, *p < 0.05

Variables like religion, place of residence, type of family, per capita income, ART status, perception of family planning method being harmful, sexual partner other than the spouse, currently sexually active, desiring another child in future, spouse desiring the same number of children, any illness in last one month showed insignificant results

As a study output, Standard Operating Procedures (SOP) for providing linked HIV and FP services were developed with a consensus of a team of experts representing program managers, international NGOs and academicians to synthesize technical guidance for use by the programs. SOPs provided a brief overview and stated convergence strategy details of operationalizing linkage between HIV/STI and FP facilities. Job descriptions were detailed out for various healthcare providers at both ART and FP centers. Training modules with various case studies, role models, and pre- and post-assessment forms were generated. The WHO eligibility criteria wheel was described in detail. The essential supply of drugs and commodities along with MIS was elaborated eloquently to strengthen monitoring of services.

## Discussion

Contraceptive use in India is skewed with majority women in reprodutive age group resorting to sterilization and only 10% using condoms. However, when it comes to PLHIV the skew is reversed such that almost all resort to using only condoms and other methods are rarely used. The unmet need of contraception among PLHIV is not distinctly different to that of the general population. A recent Indian study at ART center in an urban tertiary hospital reported an unmet family planning need at 17% among PLHIV women [11]. Reliance on less effective methods such as condom alone moreover predisposes PLHIV to the risk of unfavorable outcomes such as an unintended pregnancy.

This study implemented a strategy linking HIV and FP services to improve use of dual contraception methods among disadvantaged PLHIV group. The study cohort primarily composed of socio-economically deprived subsections of the PLHIV population. The majority in this cohort were in unskilled occupations and had low education and income levels. Nearly half the participants were from urban slums or rural regions. Association of such socio-economic determinants to non-adherence of ART treatment services in spite of universal coverage of services has been documented in varied settings suggesting the need for support measures beyond treatment services [23, 24]. In this study, we have demonstrated feasibility and effectiveness of linking HIV and FP programs by counseling and referral mechanisms to improve FP uptake by PLHIV. Majority (87%) of the enrolled couples in this study reached FP centers as a result of the intervention. With zero participants using dual methods at enrollment, this study showed significant improvement uptake of 44% acceptance by end of the six follow up months as a result of motivation and promotion of dual methods. Our previous study at two urban tertiary hospitals showed a 33% uptake of dual methods [17]. This present study additionally included couple counseling and linkage referral services in the intervention package compared to the previous study which provisioned contraception methods to only PLHIV women. Both our studies from varied settings in India have reported a higher acceptance and is higher than a similar study from Tanzania (Africa) showing an 18% improvement in acceptance of dual methods [25]. Our study findings also report improved acceptance of long-term contraceptives among dual method users, in line with similar evidence from Thailand. This may be attributed to contraceptive effectiveness and low associated risks [26]. Oral contraceptives, IUCDs and tubal ligation were found to be the most commonly accepted dual method in our study with good continuation rates. Our study contradicts dual hypothesis theory that suggests condom use reduces if a PLHIV uses more effective contraception [27]. After the intervention, condom use in our study improved and remained consistently high throughout the study. The seven unplanned pregnancies in our study were addressed early by counseling to ensure access to further medical care. Subsequently, almost all unplanned pregnancies underwent medical termination of pregnancy as per the desires of the women after taking an informed decision. This was followed by counseling and acceptance of post-abortion contraception by all women.

Studies conducted in African context have shown HIV–FP integration or linkage interventions to be successful in terms of health outcomes and cost-effectiveness, but evidence for India in this case remains limited. India has a relatively low HIV prevalence among the general population. A bidirectional linkage between HIV and FP services for India would be cumbersome and expensive given the large-scale resource investment needed for such a strategy and India’s declining HIV prevalence. The current PPTCT program in India is limited by way of its pregnant woman centricity. Guidelines focus on provisioning of a therapeutic regimen to HIV infected pregnant women while less emphasis is laid on prevention of an unwanted pregnancy itself. The use of dual methods of contraception widely ensures better protection against unintended pregnancies, HIV and other sexually transmitted infections. PLHIV like other non-infected women may not be eager to practice contraception and base decisions on various contextual factors such as spouse, family, desires, etc. A systematic review evaluating studies focusing on integration programs across sub-Saharan Africa has observed the need for integration efforts especially in low contraceptive use prevalence to address community-wide and HIV specific barriers in using FP methods along with improvement of access to information, commodities, and services within routine care [28]. Our study has focused on improving such intangible intricacies within the healthcare system to ensure comprehensive FP services to PLHIV by linking mechanisms. Provider aspects such as easy hospital registration procedure, delivery of comprehensive counseling services, training, providing available clear guidelines on medical eligibility of contraception use, sensitization to prevent stigma and discrimination have been addressed in this study which needs to be factored in decision making for the diverse Indian public health system.

This study recognizes the unidirectional linkage mechanism to be a feasible strategy to improve health outcomes that can potentially contribute to the Indian national program realizing its aim of convergence and mainstreaming of HIV into general health services.

In Phase IV of the National AIDS Control Program (NACP), India is striving to achieve the target of zero new infections. To achieve this, many preventive strategies have been planned but emphasis on unintended pregnancy prevention is missing thus far. With emergence of newer challenges for universal coverage of curative programs, it is imperative to simultaneously focus on cost-effective preventive measures. Studies from resource-constrained settings have demonstrated a reduction of as much as 16% unintended pregnancies among HIV-infected women to potentially reduce HIV-positive birth rates equivalent to that of the on-going PPTCT effort [28]. This coupled with a range of health benefits to women and their families can improve PLHIV health outcomes [29]. Integrated services are more efficient than vertical service provisioning [30]. Though NACP has successfully contributed to keeping the overall HIV prevalence rate in India below 1%, it faces newer challenges such as scaling up of interventions and an increase in number of infections in low prevalence states [31]. NACP has outlined a thrust on convergence and mainstreaming of HIV into general services [32]. Prospectively, undertaking India specific economic evaluation studies for linkage interventions can strengthen evidence basis for upholding reproductive and sexual rights of the vulnerable PLHIV population.

### Limitations

The effectiveness of this linkage strategy was seen in a well monitored situation. Sustenance of such an intervention at a large scale with implementation of monitoring mechanisms like MIS indicators for unmet need of contraception, referrals and acceptance by all stakeholders in the healthcare delivery system may present with its own unique challenges.

## Recommendations

Directives or SOPs for implementation of linked services between HIV and Family planning services need to be designed to strengthen programmatic response. Supportive supervision and sustained program response must be maintained to ensure documentation of crucial information on family planning services, especially for the marginalized PLHIV population. Convergence between HIV and FP planning programs must be streamlined by ensuring ease of registration through common access to both services, developing standard referral mechanism using referral slips, developing IEC materials for advocacy of dual methods, dual protection and motivation of PLHIV to access services. Service providers need to be trained and sensitized to cater to the needs of PLHIV population and male counterparts need to be involved in FP method acceptance.

## Conclusion

This study illustrates unmet need of contraception among the vulnerable PLHIV population. The study demonstrates feasibility of providing linked HIV and FP services at district hospital settings accessed by socio-economically vulnerable sections. The SOPs developed from this study can be used to upscale implementation in program settings. Dual contraception use among PLHIV in fact improves consistent condom uptake and reduces unintended pregnancies as compared to exclusive condom use. These interventions must be supported with repeated counseling and monitoring of program with well-defined MIS indicators. Training and motivation of staff is an essential component to provide holistic care to PLHIV. These interventions can help strengthen Prong 2 of the PPTCT program to help achieve the target of HIV elimination.

## Data Availability

The data that support the findings of this study are available from National AIDS Control Organization, New Delhi, India but restrictions apply to these data, which were used under license for the current study, and so are not publicly available. Data are however available from the authors upon reasonable request and with permission of National AIDS Control Organization, New Delhi, India.

## Abbreviations

HIV: Human Immunodeficiency Virus
PLHIV: People Living with HIV
ART: Anti-Retroviral Therapy
OBGY: Obstetrics and Gynecology
NIRRH: National Institute for Research in Reproductive Health
AIDS: Acquired Immunodeficiency Syndrome
NACO: National AIDS Control Organization
ICMR: Indian Council of Medical Research
MDACS: Mumbai District AIDS Control Society
MSACS: Maharashtra State AIDS Control Society
FP: Family Planning
STI: Sexually Transmitted Infection
OC Pill: Oral Contraceptive Pill
IUCD: Intra-Uterine Contraceptive Device
CD4: Cluster of Differentiation 4
WHO: World Health Organization
PPTCT: Prevention of Parent to Child Transmission
TB: Tuberculosis
UN: United Nations
SRH: Sexual and Reproductive Health
MIS: Management Information System
ICTC: Integrated Counseling and Testing Center
IEC: Information, Education and Counseling
INR: Indian Rupee
USD: United States Dollar
IQR: Interquartile Range
NGO: Non-Governmental Organization
MTP: Medical Termination of Pregnancy
TL: Tubal Ligation
OPD: Out-Patient Department
OR: Odds Ratio
CI: Confidence Interval
NSV: Non-scalpel Vasectomy
SOP: Standard Operating Procedure
NOC: No Objection Certificate
NACP: National AIDS Control Programme
